# P53 and taurine upregulated gene 1 promotes the repair of the DeoxyriboNucleic Acid damage induced by bupivacaine in murine primary sensory neurons

**DOI:** 10.1080/21655979.2022.2048985

**Published:** 2022-03-10

**Authors:** Luying Lai, Yongwei Wang, Shenghui Peng, Wenjing Guo, Fengxian Li, Shiyuan Xu

**Affiliations:** a Department of Anesthesiology, Zhujiang Hospital, Southern Medical University, Guangzhou, Guangdong Province, China; b Department of Rehabilitation, Zhujiang Hospital, Southern Medical University, Guangzhou, Guangdong Province, China

**Keywords:** P53, TUG1, DNA damage, bupivacaine, dorsal root ganglion

## Abstract

The research aimed to explore the biological role of p53 protein and long non-coding RNA (lncRNA) taurine upregulated gene 1 (TUG1) in bupivacaine (bup)-induced neurotoxicity. Our work treated dorsal root ganglion (DRG) cells with bup, detected cell viability through CCK-8, apoptosis through TUNEL assays, DeoxyriboNucleic Acid (DNA) damage through γ-H2AX protein and comet assay, including p53 mRNA, protein and TUG1 expression through q-PCR and western blot, furthermore, cell viability and DNA damage were determined after the silencing of p53 and TUG1, biological information and TUG1 FISH combined with p53 protein immunofluorescence (IF) was performed to determine the cellular localization of these molecule. *In vivo* experiments, we explored the impact of intrathecal injection of bup on p53 mRNA and protein, TUG1, γ-H2AX protein expression. The results showed that bup was available to signally decreased cell viability, promoted apoptosis rate and DNA damage, additionally, bup increased p53 mRNA and protein and TUG1 expression. P53 siRNA and TUG1 siRNA significantly increased DNA damage. Furthermore, bioinformatics analysis and colocalization experiments revealed that the p53 protein is a transcription factor of TUG1, in vivo experiment, intrathecal injection of bup increased the p53 mRNA, p53 protein, TUG1 and γ-H2AX protein in the murine DRG. In this study, it was found p53 and TUG1 promote the repair of the DNA damage induced by bup in murine dorsal root ganglion cells, suggesting a new strategy for the amelioration of bup-induced neurotoxicity.

## Graphical Abstract

**Figure:**
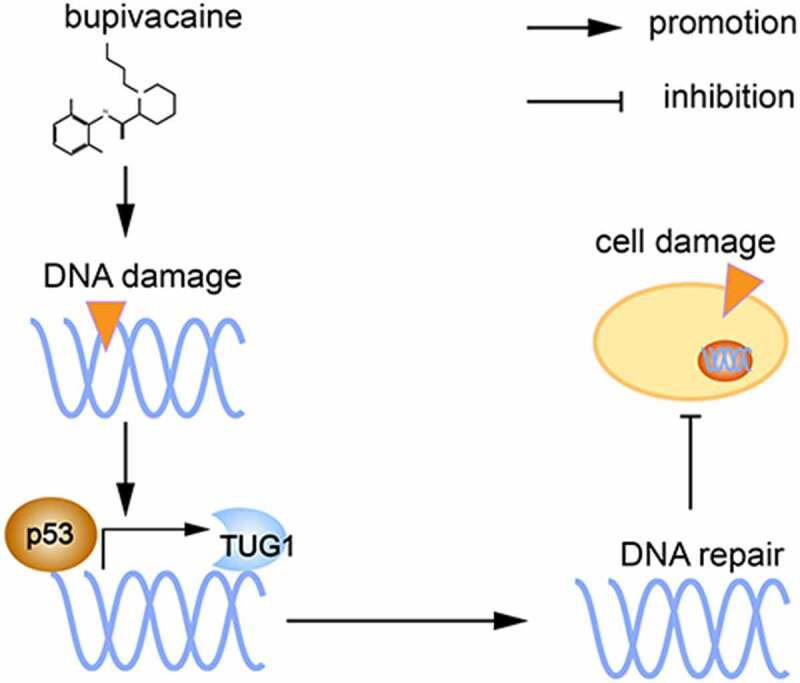

## 1. Introduction

Since the introduction of local anesthetics, they have been widely used in the clinic and have greatly promoted the rapid development of clinical anesthesia and surgery. However, numerous clinical research studies [1,2] and basic research studies [3,4] have shown that local anesthetics can cause nerve damage. The interspinal neurotoxicity of local anesthetics is mainly characterized by transient neurological syndrome (TNS), cauda equina syndrome (CES) and other poorly defined symptoms of neurotoxicity, including mental and behavioral abnormalities [5]. The disease incidence of TNS is close to 8.5% [6], and one-third of patients with TNS have severe symptoms [7]. The incidence of persistent neurological complications after spinal anesthesia is between 0.01% and 0.7% [8]. The highest incidence of severe complications occurs after various procedures are performed under local spinal anesthesia [9]. In recent years, scientific research has explored the mechanism by which local anesthetics induce neurotoxicity [10–12], but the precise mechanism remains unclear. The dorsal root ganglion (DRG), as the cell body of an axon, is a kind of primary sensory neuron located in the intervertebral foramen; hence, the DRG is the key point of signal transmission of peripheral nerve impulses to the central nervous system; thus, the DRG is the target of local anesthetics during spinal anesthesia.

The most common amide-based anesthetic used for spinal anesthesia is bupivacaine (bup), and its neurotoxicity has been attracting increasing interest [13–16]. Our previous studies have demonstrated that bup can cause DeoxyriboNucleic Acid (DNA) damage in the murine DRG [17], but the complicated mechanism underlying bup damage has not been reported [17]; therefore, the molecular mechanism underlying this DNA damage is worthy of further study.

The genome is frequently subjected to noxious endogenous or exogenous stimulation, resulting in damage; however, the genome can still maintain relative stability [18,19] because of important biological mechanisms that promote this stability in living organisms, such as the DNA damage response (DDR) [20–22], which promotes the self-healing of the damaged DNA. The p53 protein, which is a transcription factor, is involved in DNA damage repair and plays a critical role in the DDR [23,24]. Under the stress conditions caused by impaired genome integrity, p53-mediated cell cycle arrest, DNA repair, apoptosis promotion, and aging each plays a role as a ‘genome guardian’ by repairing damaged DNA or clearing irreparably damaged cells [25,26]. P53 directly regulates hundreds of RNA polymerase II transcripts and indirectly regulates thousands of genes [27] and thus participates in the development of organisms, growth, cancer progression, cellular damage responses, cell senescence, and apoptosis [28–33].

The long noncoding RNA (lncRNA) of taurine upregulated gene 1 (TUG1) has a polyadenylation tail, and the TUG1 gene is essential for the regulation of the normal development of the retina and nervous system [34]. Studies have shown that the TUG1 promoter contains a conserved p53-binding site [35], and some scholars have reported that p53-regulated TUG1 expression regulates growth during tumors development, diabetic nephropathy, and nervous system development [35].

Hence, we wondered whether the p53/TUG1 pathway is involved in bup-induced DNA damage repair in DRG cells, and we hypothesized that bup induces DNA damage and activates cell death; however, these effects are accompanied by p53 protein activation and regulation of the promoter of the downstream gene TUG1, the expression of which promotes DNA damage repair and cell survival. Our results for the first demonstrated that p53/TUG1 alleviated neurotoxicity by regulating the DNA repair, which might provide a new strategy for preventing bup-induced neurotoxicity.

## 2. Materials and methods

### 2.1 Animals

C57BL/6J mice were used in all the experiments. All the mice were purchased from the Experimental Animal Center of Southern Medical University (Guangdong, China). Three-to four-week-old female mice were used to harvest cells for primary cell culture, while mature mice were used in the animal experiments. All of the animal procedures were approved by the Institutional Animal Care and Use Committee at the Zhujiang Hosipital of Southern Medical University (Laboratory Animal Ethics Approval No. LAEC-2019-009) and were performed in accordance with the latest guidelines of the National Institutes of Health Guide for the Care and Use of Laboratory Animals.

### 2.2 DRG dissociation and culture

Three- to four-week-old C57BL/6J female mice were sacrificed with CO_2_. The DRGs were collected under a microscope with microinstruments. This process was carried out on the icebox. The separated DRGs were places in complete medium (DMEM/F12 medium supplemented with 10% fetal bovine serum and 1% penicillin-streptomycin) in an EP tube that was placed on ice, and the tissues were digested into individual cells with enzymes (0.1% Collagenase type I combined with 0.3% dispase type II) (Sigma, USA) in 37°C water bath for 30 minutes. After termination of the digestion with medium supplemented with 10% fetal bovine serum, the cell suspension was centrifuged with 15% bovine serum albumin (BSA) as the purification column, resuspended and plated in cell culture plates for further experiments. The cells were maintained in DMEM/F12 medium supplemented with 10% fetal bovine serum, 1% penicillin-streptomycin and 20 ng/ml nerve growth factor as previously described [17]. The incubator conditions were 37°C and 5% carbon dioxide. Bup was dissolved in DMEM/F12. The control group was incubated in DMEM/F12.

### 2.3 Cell counting Kit-8 (CCK-8) assay

DRG cells were cultured in 96-well plates, and each group had 6 replicate wells. After each group was treated, cell viability was measured with a CCK-8 assay kit (Tongren Institute of Chemistry, Japan) according to the protocol provided by the manufacturer. In brief, 10 μl CCK-8 solution was added to each well as previously described [17] after the addition of 100 μl DMEM/F12 solution. After incubation for 4 hours in a cell incubator in the dark, the optical density (OD) was measured at 450 nM with a microplate reader, and the OD was used to calculate the cell viability. Cell viability (%) = (OD experimental cell-OD blank)/(OD control-OD blank) ×100%.

### 2.4 Terminal deoxynucleotidyl transferase dUTP nick end labeling (TUNEL)

Bup-induced DRG neuronal apoptosis was characterized according to the method described previously [36]. An in-situ Cell Death Detection kit (catalog number: 11,684,795,910; Roche) was utilized to evaluate the apoptosis rate of DRG cells. Briefly, DRG explant was washed with PBS (Gbico, American) and quickly fixed by 2% paraformaldehyde (solarbio, Beijing, China) for 60 min at room temperature. The cells were treated with 0.1% Triton X-100 (Sigma-Aldrich, American) and cultured with TUNEL reaction mixture at 37°C for 1 h. For each experimental condition, the number of apoptotic DRG neurons was quantified as the percentage of TUNEL-positive DRG cells against DAPI cells. Fluorescent images were taken using an inverted confocal microscope (Zeiss, American).

### 2.5 Alkaline comet assay

The comet assay, also known as single-cell gel electrophoresis (SCGE), is a sensitive method for detecting DNA damage in individual cells, and the procedure was described in previous studies [37,38]. In brief, after cells were treated in the indicated manner, 10 μl of prepared cells (approximately 10^6^ cells/ml) was added to 100 μl of Low melting point agarose (LMPA) that had be pre-melted at 38°C, and the mixture was added to comet slide. After the LMPA was polymerized, the whole slide was covered with 1% agarose gel that had been melted at 56°C. The samples were incubed in a refrigerator at 4°Cfor 20 minutes, and then, the comet slide was incubated in a 4°C precooled high-salt solution for 2 hours to separate the protein from the DNA. Both DNA denaturation and electrophoresis were performed in a strong base environment (pH>13). Neutralization was carried out with precooled PBS, and then DNA staining was carried out with PI. The whole procedure was carried out under low light conditions. After cell lysis, DNA denaturation, gel electrophoresis and PI staining, small DNA fragments were broken from DNA and formed the tail structures of the comets, while the DNA body formed the structure of the comet head. Images were captured under the fluorescence microscopy. CASP 6.0 software was used to analyze the related indexes of the comet experiment, such as percentage of head DNA%, the percentage of tail DNA, and the olive tail moment.

### 2.6 Western blotting

The procedure was carried out as previously described [39]. Protein form cells or tissues was extracted with RIPA lysis buffer (Sigma, America). The protein concentration was measured with a BCA Protein Assay Kit (Tiengen Biotech, China). When polyacrylamide gel electrophoresis was completed, the proteins were electrophoretically transferred onto PVDF membranes (Bio-Rad, America). The membranes were blocked with 5% BSA for 1 hour at room temperature. The primary antibodies included rabbit anti-53 (Cell Signaling Technology, 1:1000), rabbit anti-γ-H2AX (Cell Signaling Technology, 1:1000), and mouse anti-β-actin (Cell Signaling Technology, 1:5000). The membranes were incubated in the primary antibody solutions on a shaker at 4°C overnight. The secondary antibodies used were goat anti-rabbit (BiboBio, 1:5000) and goat anti-mouse (BiboBio, 1:5000) antibodies. The extra primary antibody was washed off, and then, the membrances were incubated with the secondary antibodies for 1 h at room temperature. Then, the membranes stained with the antibodies and proteins were incubated with Western Chemiluminescent HRP Substrate (Bio-Rad, America). The level of protein expression was detected by chemiluminescence (Tanon5500), and the gray values of the protein bands were analyzed by ImageJ. The level of protein expression is expressed as expression level relative to that of the internal reference protein.

### 2.7 Total RNA extraction and qPCR analysis

Total RNA was extracted with RNAiso Plus (Takara, China), the concentration of RNA was detected with a NanoDrop 2000 (Thermo, America), and the A260/A280 ratio was used to determine the RNA purity. The PrimeScript™ RT reagent Kit (TaKaRa, China) was used for RNA reverse transcription, and the reagent used in the real-time quantitative polymerase chain reaction (qPCR) was SYBR® Premix Ex Taq™ II (TaKaRa, China) [3]. The internal reference gene used was β-actin. The primers were synthesized by Sangon Biotech company. The primer sequences are shown in Table 1. The reaction was performed with a LightCycler® 480 II (Roche, Switzerland). All of the procedures were performed according to the manufacturer’s protocol. The RNA expression levels were calculated with the Δct method.

**Table 1.** The primer sequences for qPCR

Gene	Direction	Sequences (5’-3’)
p53	p53-Forward	TGAAACGCCGACCTATCCTTA
	p53-Reverse	GGCACAAACACGAACCTCAAA
TUG1	TUG1-Forward	CATAGTATCATCTTCGGGTTAC
	TUG1-Reverse	CACAAAATGCATGTAGGTTC
β-actin	β-actin-Forward	GTGCTATGTTGCTCTAGACTTCG
	β-actin-Reverse	ATGCCACAGGATTCCATACC

### 2.8 SiRNA synthesis and transfection

For both p53 and TUG1, we synthesized 3 siRNA sequences (Sangon Biotechnology), as shown in Table 2, for screening and use. After DRG cells were plated and cultured with normal medium for 24 h, we began to transfect the siRNAs into the cells. The siRNAs were transfected into the DRG cells with Lipofectamine 3000 (Life Technology, China) and Opti-MEM (Gibco, USA) without any additional components. All of the procedures were performed according to the manufacturer’s protocol and previous report [3]. After 24 hours, the medium was replaced with normal medium, and after more than 24 hours, we began to treat the cells as planned.

**Table 2.** The target sequences of siRNA

Gene	**Target sequence**
p53 siRNA-001	TAACTCTAAGGCCTCATTC
p53 siRNA-002	CCCAGCGAAATTCTATCCA
p53 siRNA-003	GAGTCACAGTCGGATATCA
TUG1 siRNA-001	CCATCTCACAAGGCTTCAA
TUG1 siRNA-002	CATATTGTCAACCGTTT
TUG1 siRNA-003	TCAGTTTCAGCCTCTCCTT

### 2.9 Retrieval of relationship about the relationship between p53 and lncRNA TUG1

To determine whether the p53 protein is a transcription factor of TUG1, we retrieved information about the relationship between p53 and TUG1. In particular, we obtained information about the location, promoters, enhancers and gene enhancer regulatory elements for the TUG1 gene from https://www.genecards.org/cgi-bin/carddisp.pl?gene=TUG1&keywords=TUG1. In addition, to verify this result, we also conducted related searches in the Promo database. First, we searched for the promoter of the murine TUG1 gene (2,000 bases upstream of TUG1) in http://genome.ucsc.edu/. Secondly, we input the TUG1 promoter sequence in the search bar of the Promo Database (http://alggen.lsi.upc.es/cgi-bin/promo_v3/promo/promoinit.cgi?dirDB=TF_8.3), and set the maximum matrix dissimilarity rate to 5%.

### 2.10 LncRNA TUG1 and p53 protein colocalization experiment: TUG1 FISH combined with p53 protein immunofluorescence (IF)

If the p53 protein promotes the expression of lncRNA TUG1, p53 would bind to the promoter of TUG1, so we used FISH combined with P53 protein experiment to verify whether there is a colocation relationship between these molecules [40]. We obtained a mouse TUG1 probe FISH kit from RiboBio, and the FISH experiment was carried out according to the manufacturer’s protocol. All the reagents and consumables were enzyme-free. In brief, cells were fixed with 4% paraformaldehyde for 10 minutes at room temperature. Then, the cellular membrane structure was permeabilized with 0.5% Trito X-100, and the cells were blocked with a mixture of prehybridization buffer and blocking buffer for 30 minutes at 37°C. The cells were washed with PBS for 3 times for 5 mins each time. Then, the pre-hybridization mixture was removed, the TUG1 probe solution formulated with hybridization buffer was added, and the cells were incubated in a cell culture incubator in the dark overnight. The fluorescein of the labeled probe was excited to emit red fluorescence, and the remaining part of the experiment was carried in a low light environment. After incubation with the TUG1 probe, the cells were blocked with 5% BSA for 1 hour at room temperature, incubated with a p53 primary antibody (CST, USA) at 4°C overnight, washed the next day, incubated with a fluorescent secondary antibody (goat anti-rabbit, CST, USA) that could emit green fluorescence, mounted with DAPI, placed on a glass slide and observed under a fluorescence microscope.

### 2.11 Intrathecal injection

We injected 10 μl of NS (NS group) or the corresponding concentration of bup (0.5% bup group or 0.75% bup group) into the L_3_-L_4_ subarachnoid space in each mouse to simulate human spinal anesthesia. The method of intrathecal injection was performed as previously described [41]. The indications of the successful intrathecal injection of bup were that the motor function of the lower limbs of the mice was inhibited, that the limb remained in a towed state, and that the motor function of the lower limbs of the mice could be restored within 30 minutes. No change in the motor function was observed in the control mice injected with normal saline. Thirty minutes after the injection, the mice were killed, the DRGs below T_10_ were isolated, the protein or RNA was extracted for corresponding assessment.

### 2.12 Statistics

The measurement data in this study are expressed as the mean ± standard deviation ($\bar{x}$ ± *s*). GraphPad Prism 5 software was used for data analysis by Student’s t test detection and one-way analysis of variance (ANOVA). Tukey’s test was used to make multiple variance corrections to the data. At least 3 biological replicates were performed per experiment, and *P* < 0.05 indicated that the difference was statistically significant.

## 3. Results

In this study, we aimed to explore the role and molecular mechanism of p53 and TUG1 in bup-induced neurotoxicity. A series of i*n vitro* assays were conducted, and the results indicated that p53/TUG1 exhibited neuroprotective effects against neurotoxicity. Mechanistic investigations revealed bup-induced DRG DNA damage, and then trigger DAN repair by regulating p53/TUG1 pathway, which alleviated bup-induced neurotoxicity.

### 3.1 Bup caused DRG cell damage by reducing cell viability and promoting apoptosis.

**Figure 1.**
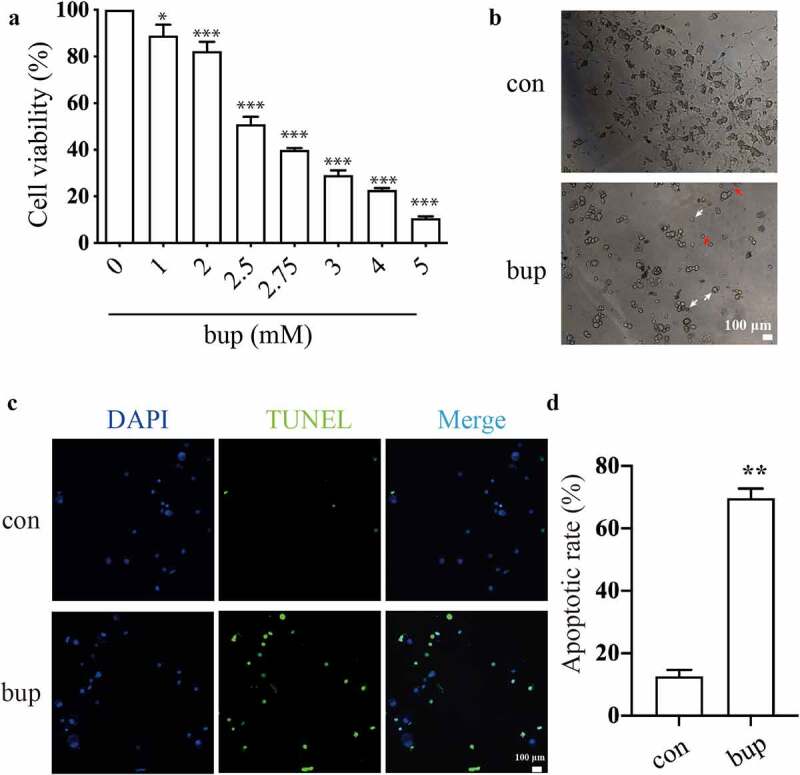
**Bup caused DRG cell damage**. (a)**: Bup decreased DRG cells viability in concentration dependent manner**. After DRG cells were treated with different concentrations (0, 1, 2, 2.5, 2.75, 3, 4, 5 mM) of bup for 3 h, CCK-8 assays were performed (n = 6), One-way ANOVA with Tukey’s test, *vs* con, *P < 0.05, ***P < 0.001. (b): Representative images shown cell injuries after bup challenges. Red arrows indicated broken filaments, white arrow heads shown shrunk cell bodies. **(c&d): Bup increased the ratio of apoptosis cells**. DRG neurons were isolated from mouse and then cultured in media with or without 2 mM bup, the numbers of cells in apoptosis were measured via TUNEL (green) staining, green fluorescence indicates apoptosis cells; the analysis graph for the ratio of apoptosis cells (n = 3), Student’s T test, *vs* con, **P < 0.01.

First, we treated the detached DRG cells with different concentrations of bup (0, 1, 2, 2.5, 2.75, 3, 4, and 5 mM) for 3 h, and the cell viability assays showed that bup treatment decreased cells viability in a concentration-dependent manner (Figure 1 A), which was consistent with our published research [42]. In the present experiment, we found that when the concentration of bup was equal to or greater than 2.5 mM, the cells easily detached from the culture plates; therefore, 2 mM bup was used in later experiments. Indeed, after bup challenge, sensory neurons changed their morphology into a shrunk size and presented with broken filaments (Figure 1b).

In addition, the TUNEL assay showed that compared with that in the control group, the ratio of apoptotic cells in the bup group was significantly increased, which indicates that the cells in the bup groups underwent apoptosis at a dramatically higher rate (Figure 1 C&D).

### 3.2 Bup exacerbated DNA damage in DRG cells.

**Figure 2.**
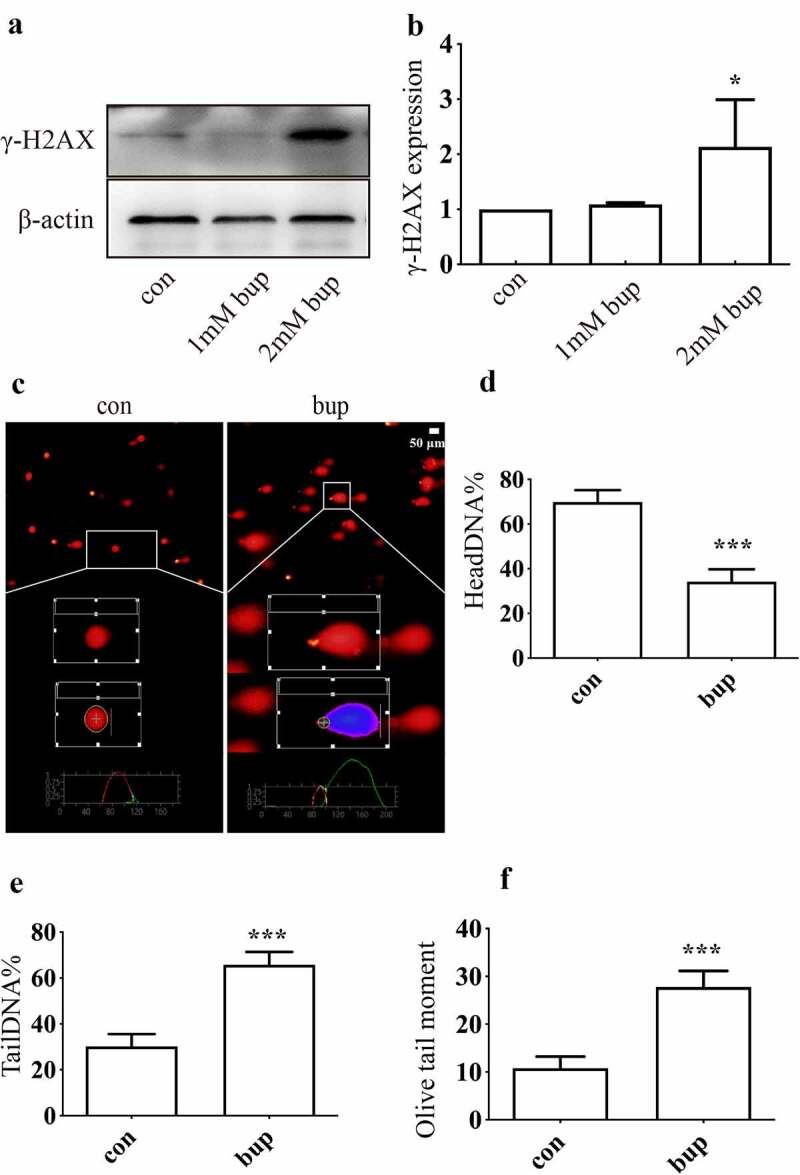
**Bup caused DNA damage in DRG cell. (A&B): Bup increased γ-H2AX protein expression**. After DRG cells treated with or without bup (1 mM, 2 mM) for 3 h, western blot detected γ-H2AX protein expression (n = 3), One-way ANOVA with Tukey’s test, *vs* con, *P < 0.05. (c-f)**: Bup induced DNA damage**. After cells were treated with or without 2 mM bup for 3 h, comet assay was complemented to test DNA damage, images show the comet track. The analysis graph for HeadDNA%, TailDNA% and Olivetailmoment were presented in Figure d-f. three biological replicates for each exposure condition were pooled and 60 random cells per slide were assessed for DNA damage (n = 3), Student’s T test analysis the result, *vs* con, ***P < 0.001.

γ-H2AX is a DNA repair protein whose expression is commonly measured to quantify double-stranded DNA damage [43–45]. Compared with that in the control group, the expression of γ-H2AX in the 1 mM bup group was not significantly different; however, 2 mM bup increased γ-H2AX protein expression (Figure 2 A&B).

The percentage of headDNA%, the percentage of tailDNA% and the olivetailmoment were used to evaluate the degree of DNA damage (Figure 2 C-F). Compared with the control group, the bup group showed a lower percentage of head DNA but a higher percentage of tail DNA and a higher olive tail moment, which suggested that the bup-treated group exhibited much more severe DNA damage than the control-treated group. All of these results demonstrate that bup can exacerbate DNA damage in DRG cells.

### 3.3 DNA damage induced by bup promotes p53 expression, and p53 expression promotes DNA repair, alleviating DNA and cell damage.

**Figure 3.**
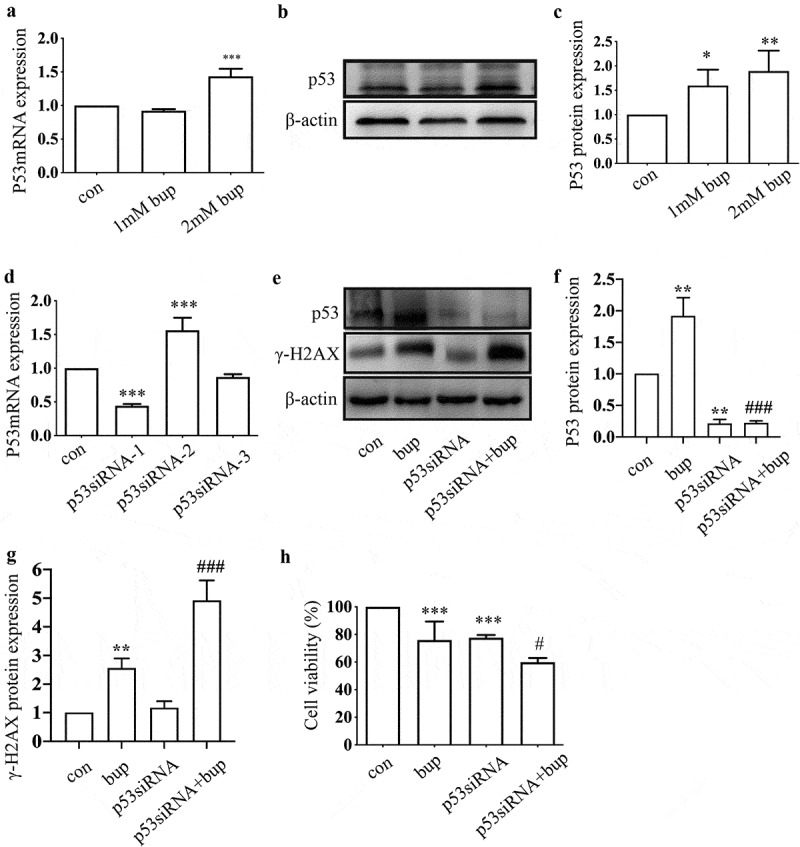
**P53 induced DNA repair to alleviate DNA and cell damage**. (a-c)**: Bup significant increased p53 mRNA and protein expression**. After cells were planted for 24 h, DRG cells were treated with 0 mM, 1 mM and 2 mM bup for 3 h, q-PCR and western blot were complemented to test p53 mRNA and protein expression (n = 3), One-way ANOVA with Tukey’s test, *vs* con, **P < 0.01, *P < 0.05, ***P < 0.001. (d)**: Q-PCR detected the silence effect of p53 siRNA-1 was best**. DRG cells were treated with p53siRNA for 24 h, Q-PCR was complemented to test p53 mRNA expression (n = 3), One-way ANOVA with Tukey’s test, *vs* con, ***P < 0.001. (e-g)**: P53 siRNA increased γ-H2AX protein expression induced by bup**. DRG cells were treated with or without p53 siRNA for 24 h, replace the medium with normal medium for 24 h, cells were treated with or without 2 mM bup, western blot was complemented to test p53 protein and γ-H2AX protein expression (n = 3), One-way ANOVA with Tukey’s test, *vs* con, **P < 0.01; *vs* bup, ###*P* < 0.01. (f)**: P53 siRNA decreased cells viability induced by bup**. DRG cells were treated as above, CCK-8 was complemented to test cell viability (n = 6), One-way ANOVA with Tukey’s test, *vs* con, ***P < 0.001; *vs* bup, #*P* < 0.05.

We aimed to determine whether p53 participates in the DDR in DRG cells treated with bup and the possible role played by p53. Compared with that in the control group, p53 mRNA expression in the 2 mM bup group was significantly higher (Figure 3A). In addition, the expression of the p53 protein was increased in 1 mM bup and 2 mM bup groups, with the higher expression in the 2 mM bup group (Figure 3 B&C). These findings indicate that the noxious stimulation of bup upregulates p53 expression.

**Figure 4.**
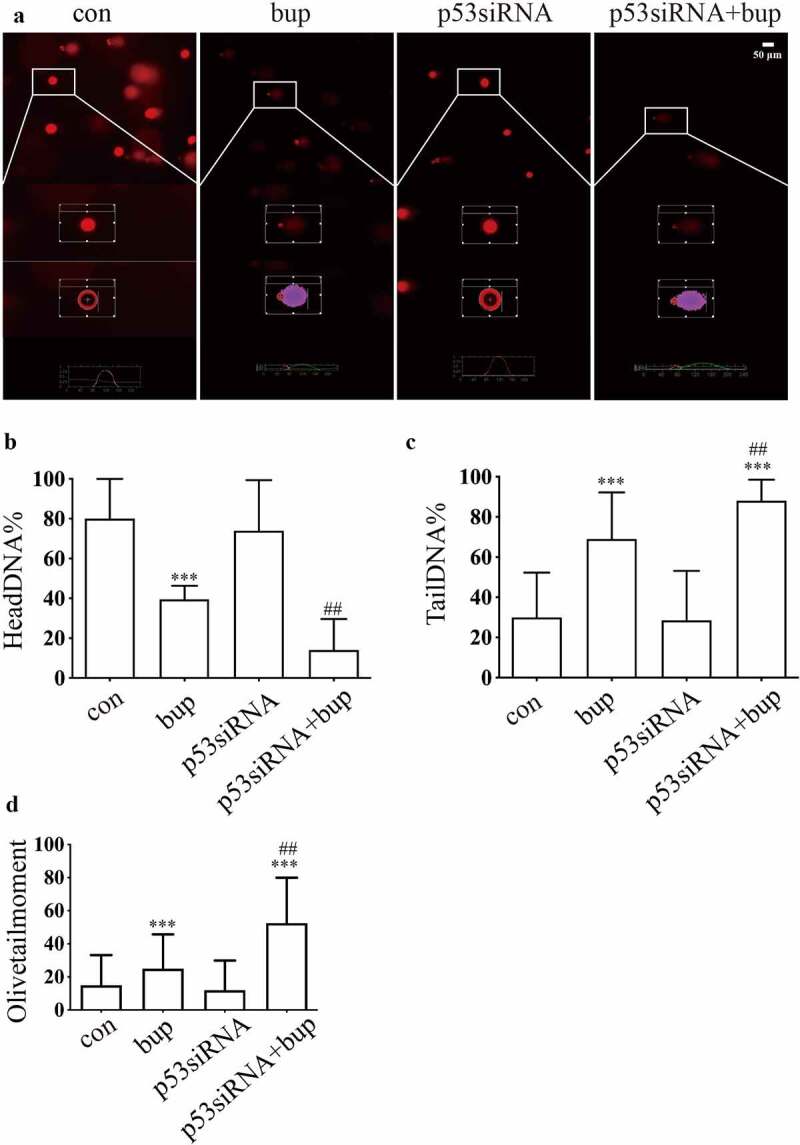
**P53 siRNA inhibited DNA repair to alleviate DNA and cell damage**. (a)**: Images show the comet track detected by Comet assay**. DRG cells were treated with or without p53 siRNA for 24 h, replace the medium with normal medium, after more 24 h, cells were treated with or without 2 mM bup. (b-d)**: The analysis graph for HeadDNA%, TailDNA% and Olivetailmoment were presented in Figure b-d**; One-way ANOVA with Tukey’s test, *vs* con, ***P < 0.001; *vs* bup, ##*P* < 0.01.

**Figure 5.**
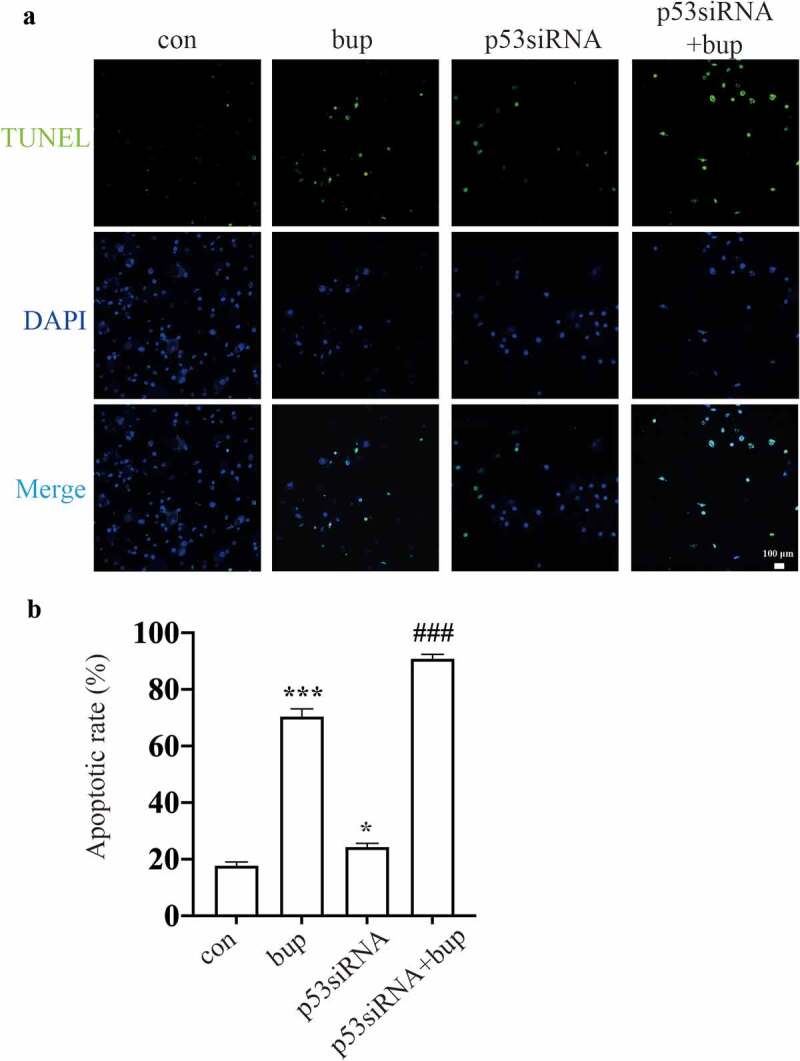
**P53siRNA increased the ratio of apoptosis cells. (A&B**): DRG cells were treated with or without p53 siRNA for 24 h, replace the medium with normal medium, after more 24 h, cells were treated with or without 2 mM bup, the numbers of cells in apoptosis were measured via TUNEL (green) staining, green fluorescence indicates apoptosis cells; the analysis graph for the ratio of apoptosis cells (n = 3), One-way ANOVA with Tukey’s test, *vs* con, *P < 0.05, ***P < 0.001; *vs* bup, ###*P* < 0.001.

Hence, we wondered whether p53 a protective factor in this process. We synthesized three p53 siRNA sequences and, after transfection, investigated the p53 mRNA silencing effect by qPCR. The results showed that p53 siRNA-1 was the most effective siRNA tested (Figure 3D); therefore, p53 siRNA-1 was used in the follow-up experiments. After transfection of the cells with p53 siRNA, the cells were treated with 2 mM bup for 3 h. Then, a CCK-8 assay was used to measure cell viability, and an alkaline comet assay was performed to assess DNA damage. The results showed that compared with that in the control group, cell viability in the treatment groups decreased. Moreover, compared with the bup treatment alone, p53siRNA+bup treatment further reduced the cell viability, which suggests that p53 may be a protective factor against bup-induced neurotoxicity (Figure 3 E). The results of the comet assay were consistent with those of the CCK-8 assay. After p53 mRNA expression was downregulated, DNA damage was further exacerbated (Figure 4 A-D), apoptosis rate also increased (Figure 5 A&B). All of these results demonstrate that the DNA damage induced by bup promotes p53 expression and that p53 promotes DNA repair to alleviate DNA and cell damage.

### 3.4 Downregulation of the lncRNA TUG1 expression affected bup-induced DRG cell viability and DNA damage in a manner similar to downregulation p53 expression

**Figure 6.**
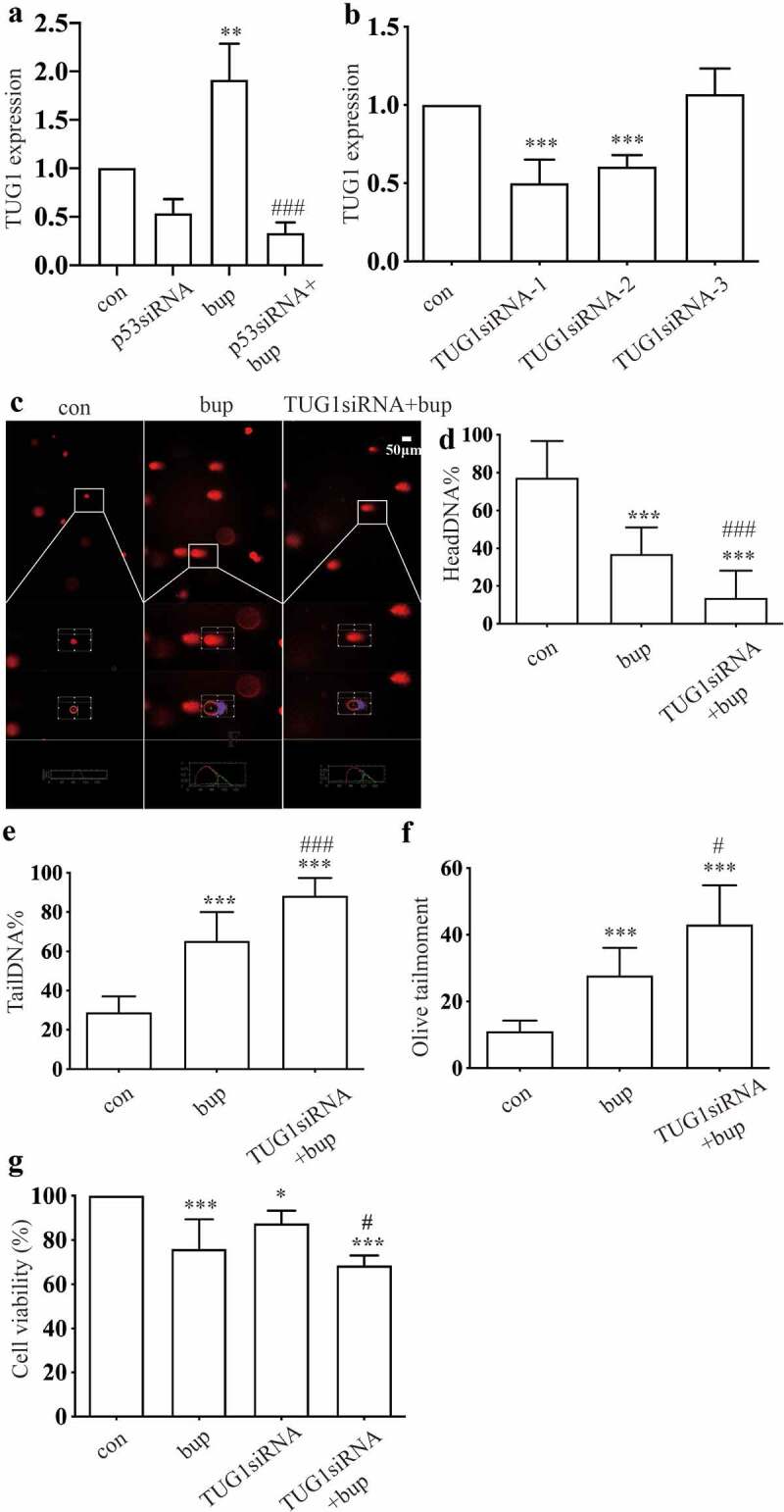
**TUG1 was a protective factor in bup induced DRG damage**. (a)**: P53 siRNA downregulated lncRNA TUG1 expression**. After cells were planted for 24 h, DRG cells were treated with p53 siRNA for 24 h, q-PCR was complemented to test TUG1 expression (n = 3), One-way ANOVA with Tukey’s test, ***P < 0.001. (b)**: Q-PCR detected the silence effect of TUG1 siRNA-1 and TUG1 siRNA-2 was best**. After cells were planted for 24 h, DRG cells were treated with TUG1 siRNA for 24 h, q-PCR was complemented to test TUG1 expression (n = 3), One-way ANOVA with Tukey’s test, *vs* con, ***P < 0.001. (c-f)**: Comet assay detected TUG1 siRNA aggravates DNA damage induced by bup**. After cells were planted for 24 h, DRG cells were treated with or without TUG1siRNA for 24 h, replace the medium with normal medium, after more 24 h, cells were treated with or without 2 mM bup, comet assay was complemented to test DNA damage (n = 3), One-way ANOVA with Tukey’s test, *vs* con, ***P < 0.001; *vs* bup, #*P* < 0.001, ###*P* < 0.001. (g)**: TUG1 siRNA decreased cells viability induced by bup**. After cells were treated as above, CCK-8 was complemented to test cell viability (n = 6), One-way ANOVA with Tukey’s test, *vs* con, **P < 0.01,***P < 0.001; *vs* bup, #*P* < 0.05.

TUG1 is an interesting gene, and we wanted to explore the function of the lncRNA TUG1 in bup-induced DRG damage. Compared with that in the control group, TUG1 expression in the p53 siRNA group was significantly decreased (Figure 6 A), which indicates that the TUG1 gene may be the downstream of p53. Moreover, the results showed that TUG1 expression was significantly increased in the 2 mM bup group (Figure 6A), showing an expression trend similar to that of the p53 expression.

Then, we used q-PCR to investigate the silencing efficiency of synthetic TUG1 siRNA (Figure 6B). The results showed that TUG1 siRNA-1 and TUG1 siRNA-2 were effective; thus, a mixture of TUG1 siRNA-1 and TUG1 siRNA-2 was used in subsequent experiments. After DRG cells were transfected with TUG1 siRNA, we added 2 mM bup to the cell culture and incubated the cells for 3 h. the comet assay showed that DNA damage was also reduced in the TUG1 siRNA+bup group (Figure 6C-F). In addition, Compared with that of the bup group, the cell viability of the TUG1 siRNA+ bup group was further diminished (Figure 6 G).These experiments showed that downregulation of TUG1 expression has effects similar to those of downregulation of p53 expression; therefore, the lncRNA TUG1 is also a factor that protects against bup- induced DRG damage.

### 3.5 Information retrieved from the databases revealed that p53 is located the TUG1 promoter.

From the GeneCards database, we obtained genomic TUG1 location information (Supplementary Figure 1 A), gene enhancer regulatory elements sequence for TUG1, and promoters and enhancers sequences for the TUG1 gene (Supplementary Figure 1 B). The results of our analyses showed that the p53 gene has a high score for banding to the promoter of TUG1. From the University of California-Santa Cruz (UCSC) database, we obtained the base sequence information of the TUG1 promoter (2000 bases upstream of TUG1; supplementary table 1). We inserted the TUG1 promoter sequence into the PROMO database, and P53 was identified in the search results (Supplementary Figure 1 C). By adding a p53 label to the results table, we identified the predicted sequence of the TUG1 promoter to which p53 binds (Supplementary Figure 1 D). The results of the information retrieval process revealed that p53 may be a transcription factor of TUG1 and that it has a theoretical binding site in the TUG1 promoter.

### 3.6 In mouse DRG cells, the lncRNA TUG1 and p53 have common expression sites.

Previous experiments showed that p53 and TUG1 have similar responses to bup-induced DRG injury, and the information retrieval process revealed that p53 is a transcription factor of TUG1. However, we wondered whether p53 is a transcription factor of TUG1 in response to bup-induced DRG damage. If it is, then the connection between TUG1 and p53 needs to be explored.

**Figure 7.**
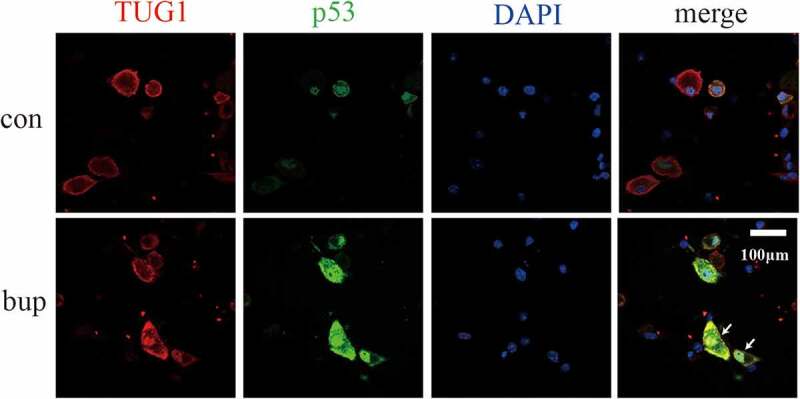
**P53 and TUG1 share a common expression site**. Blue fluorescence site is DAPI stained cell nucleus; red fluorescence is the lncRNA TUG1 expression site labeled in the FISH partial experiment; green fluorescence is the p53 protein expression site stained.

The results of the lncRNA TUG1-protein p53 colocalization experiment are shown in Figure 7. Red fluorescence indicates the sites of lncRNA TUG1, green fluorescence indicates the sites of the p53 protein, and blue fluorescence indicates the nucleus. The results showed that p53 colocalized with TUG1 in the bup group. This finding indicates that p53 and TUG1 likely colocalize after bup treatment.

### 3.7 Animal experiments showed that p53 and TUG1 expression is upregulated after bup intrathecal injection

**Figure 8.**
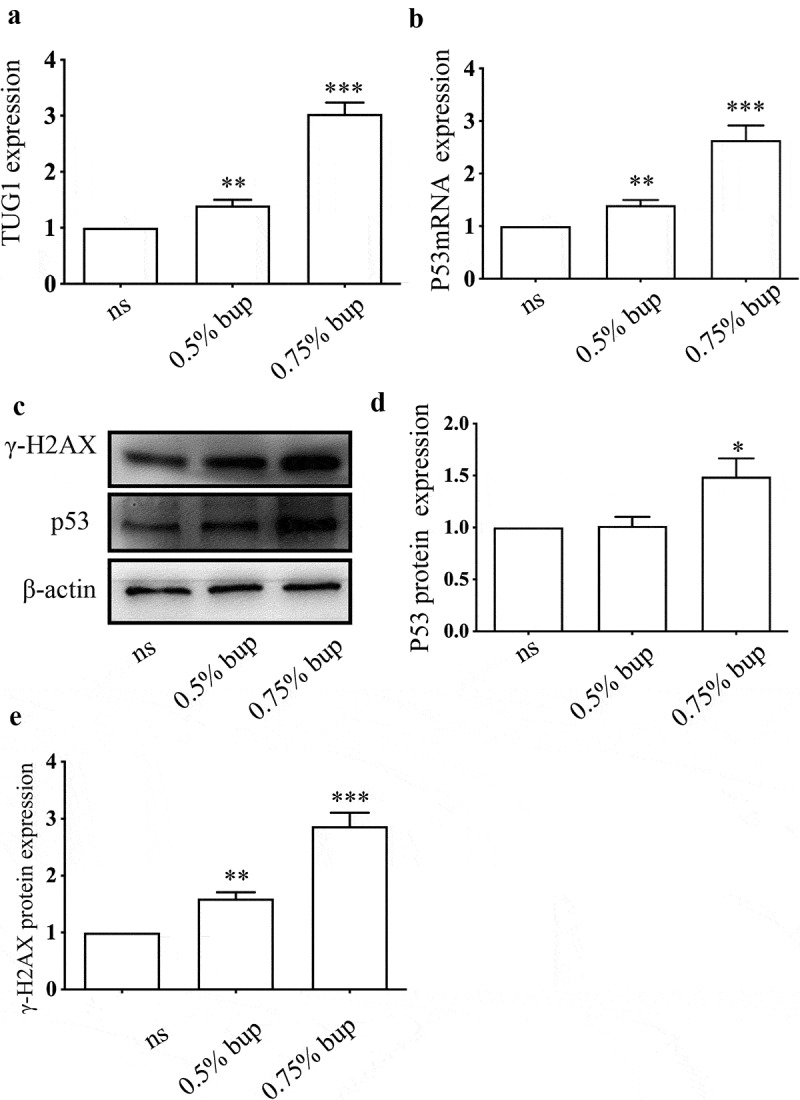
**Expression of TUG1, P53 mRNA and protein, γ- H2AX protein in DRG of lumbosacral region of mice after intrathecal injection of bup. (a&b): Bup increased TUG1 and p53 mRNA expression in DRG tissues of mice**. We injected 0.9% normal saline, 0.5% bup into the subarachnoid space of mice to simulate clinical subarachnoid anesthesia, DRG of T10-L5 were separated after 30 minutes, the expression of lncRNA TUG1 and mRNA p53 was detect by Q-PCR (n = 3), One-way ANOVA with Tukey’s test analysis, vs ns, **P < 0.01, ***P < 0.001. (c-e)**: Bup increased p53 and γ- H2AX protein expression in DRG tissues of mice**. We injected normal saline, 0.5% bup, 0.75% bup into the subarachnoid space of mice, DRG of T_10_-L_5_ were separated after 30 minutes, the expression p53 and γ- H2AX protein expression was detect by western blot (n = 3), One-way ANOVA with Tukey’s test analysis, *vs* ns, *P < 0.05, **P < 0.01, ***P < 0.001.

To explore the roles of p53 and TUG1 *in vivo*, we injected 0.9% normal saline (NS) or 0.5% bup, 0.75% bup into the subarachnoid space of mice to simulate clinical subarachnoid anesthesia. The results showed that, compared with that in the NS group, the expression of the lncRNA TUG1 and p53 mRNA was upregulated in the 0.5% bup and 0.75% groups (Figure 8 A&B); additionally, the protein expression of p53 and **γ**-H2AX protein increased in the DRG of mice after intrathecal injection with 0.75% bup (Figure 8 C-E). These results are consistent with the cell experiment results.

## 4. Discussion

Recent research has shown that all local anesthesia is toxic and that the effects of this toxicity on nerves and muscles are dose- and time-dependent [46]. The mechanism underlying local anesthetic neurotoxicity is generally complex, and researchers have tried to explain it from different perspectives [10]. The latest reports points out that bup induced neurotoxicity by regulating the miR-421/zinc finger peotein564 in SH-SY5Y cells [47], mepivacaine induced neurotoxicity by regulating the miR-183-5p in SH-SY5Y cells [48], however, our study selected primary murine sensory neuron DRG as the research model, which can better explain the toxic mechanism of bup because it is an important secondary neurons of sensory [49]. To examine the neurotoxicity of bup *in vitro*, we studied primary murine sensory neuron DRG cells and found that bup exhibited toxicity in a concentration-dependent manner. The half-maximal inhibitory concentration (IC50) of bup in DRG cells was between 2.5 and 3.0 mM. However, when the concentration of bup was more than 2.5 mM, the cells easily detached from the plates; therefore, in later experiments, bup was used at a 2 mM concentration. In previous studies, we explored the neurotoxic effects of local anesthetics from the perspective of DNA damage [42,50] and found that bup induced ku70-independent DNA damage [17]. In the present study, we further found that p53/TUG1 promotes the repair of DNA damage induced by bup in murine DRG cells.

We know that DNA carries all the genetic material of eukaryotes that controls cell growth, proliferation, differentiation, senescence, and apoptosis. Genome integrity plays an important role in the whole life cycle of cells. When any part of the cell is damaged, genes that facilitate damage repair are expressed. The perfect function of DNA plays a vital role in the biological activities of cells. Studies on DNA damage have focused on the central nervous system, but studies on peripheral nerves have been limited **[**51**]**.

P53 promotes the repair of damaged DNA in many tissues [26,52,53]. Experimental results indicated that bup induced notable DNA damage, entire cell damage and even apoptosis, and subsequently, the mRNA and protein expression of p53 increased. As p53 is mainly expressed in the nucleus and plays a role in promoting DNA repair in many types of tissues [26,54], we think p53 is also involved in the process of the bup-induced DDR. To validate this hypothesis, we downregulated p53 mRNA expression through p53 siRNA transfection and found that DNA damage and cell damage were exacerbated. The DNA damage in the p53 siRNA transfection group without bup treatment was also greater than that in the control group. We think this result was caused by the inevitable of DNA damage induced by cell treatment because DNA is easily damaged under various physical and chemical conditions [55]. This finding indicated that p53 is a protective factor in the progression of the bup-induced DRG damage response.

P53-dependent DNA repair is achieved through the regulated expression of numerous downstream genes, including the regulated expression of numerous lncRNAs **[**56,57**]**. In our experiments, TUG1 showed the same expression trend as the p53 protein. When p53 mRNA expression was reduced, TUG1 expression was also reduced, and silencing of p53 mRNA and TUG1 expression exacerbated DNA damage and cell death after the bup-induced DRG damage response was activated; therefore, we consider that TUG1 is a positive regulatory gene that is downstream of p53. Because we wondered whether TUG1 is a target gene of p53, we performed an information search in the PROMO and GENECARD databases to predict the role of p53 as a transcription factor of TUG1, and the results indicated that p53 has a specific binding site in the promoter of TUG1. Then, the lncRNA TUG1 FISH combined with a p53 protein IF assay verified that the p53 protein and TUG1 have the same expression site, which is consistent with the targeted relationship between the p53 protein and TUG1 **[**35,58,59**]**. *In vivo*, p53 mRNA, p53 protein, γ-H2AX protein and lncRNA TUG1 expression also increased after subarachnoid anesthesia with 0.5% bup treatment, which was consistent with the *in vitro* experiment. We presume that γ-H2AX is expressed during the late phase in the chain reaction of double-stranded DNA repair **[**60,61**]**. We believe that the p53 protein activates and upregulates the expression of TUG1 and promotes the repair of damaged DNA, thereby reducing DNA damage and the overall level of cell damage.

## Conclusion

**Figure 9.**
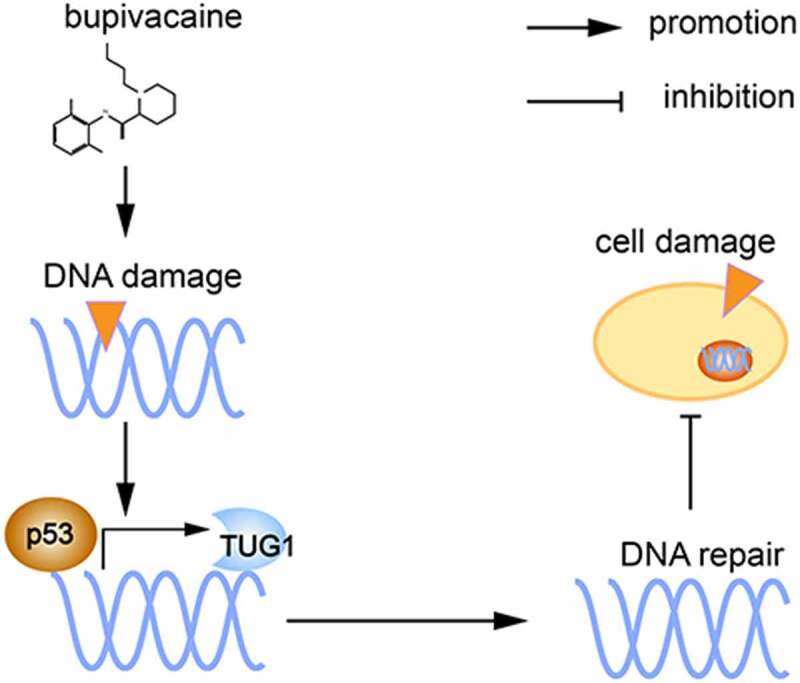
**Proposed mechanism**: bupivacaine produce DNA damage in DRG cells, p53 expression increased and promote lincRNA TUG1expression as a transcription factor, final alleviate DNA damage and thereby reduce cell damage.

Through the cell experiments described here, we demonstrated that bup induces DNA damage and affects the expression of the DNA repair-associated p53 protein. P53 protein is a transcription factor that promotes lincRNA TUG1 expression, ultimately alleviating DNA damage and reducing cell damage (Figure 9 shows the proposed mechanism). These findings suggested that p53/TUG1 could be considered as a new therapeutic target for bup-induced neurotoxicity treatment. However, this study has its limitations, for example, future studies are required to explore the direct action mechanism p53 and TUG1 *in vivo* and the precise mechannism by which TUG1 regulates DNA repair.

## Supplementary Material

Supplemental Material

## Abbreviations

Bup, bupivacaine; DNA, DeoxyriboNucleic Acid; DRG, dorsal root ganglion; IF, immunofluorescence; lincRNA, long intergenic noncoding RNA; TUG1, taurine upregulated gene 1; TNS, transient neurological syndrome; CES, cauda equina syndrome; DDR, DNA damage response; BSA, bovine serum albumin; OD, optical density; SCGE, single-cell gel electrophoresis; LMPA, Low melting melting point agarose; IC50, half-maximal inhibitory concentration.
