# Supplementary material for: P53 and taurine upregulated gene 1 promotes the repair of the DeoxyriboNucleic Acid damage induced by bupivacaine in murine primary sensory neurons
Source: Bioengineered. 2022 Mar 10;13(3):7439–56. doi: 10.1080/21655979.2022.2048985 (PMC9208530; doi:10.1080/21655979.2022.2048985)
Supplement: Supplemental Material [file KBIE_A_2048985_SM0069.zip › Supplemental Figure 1.docx]

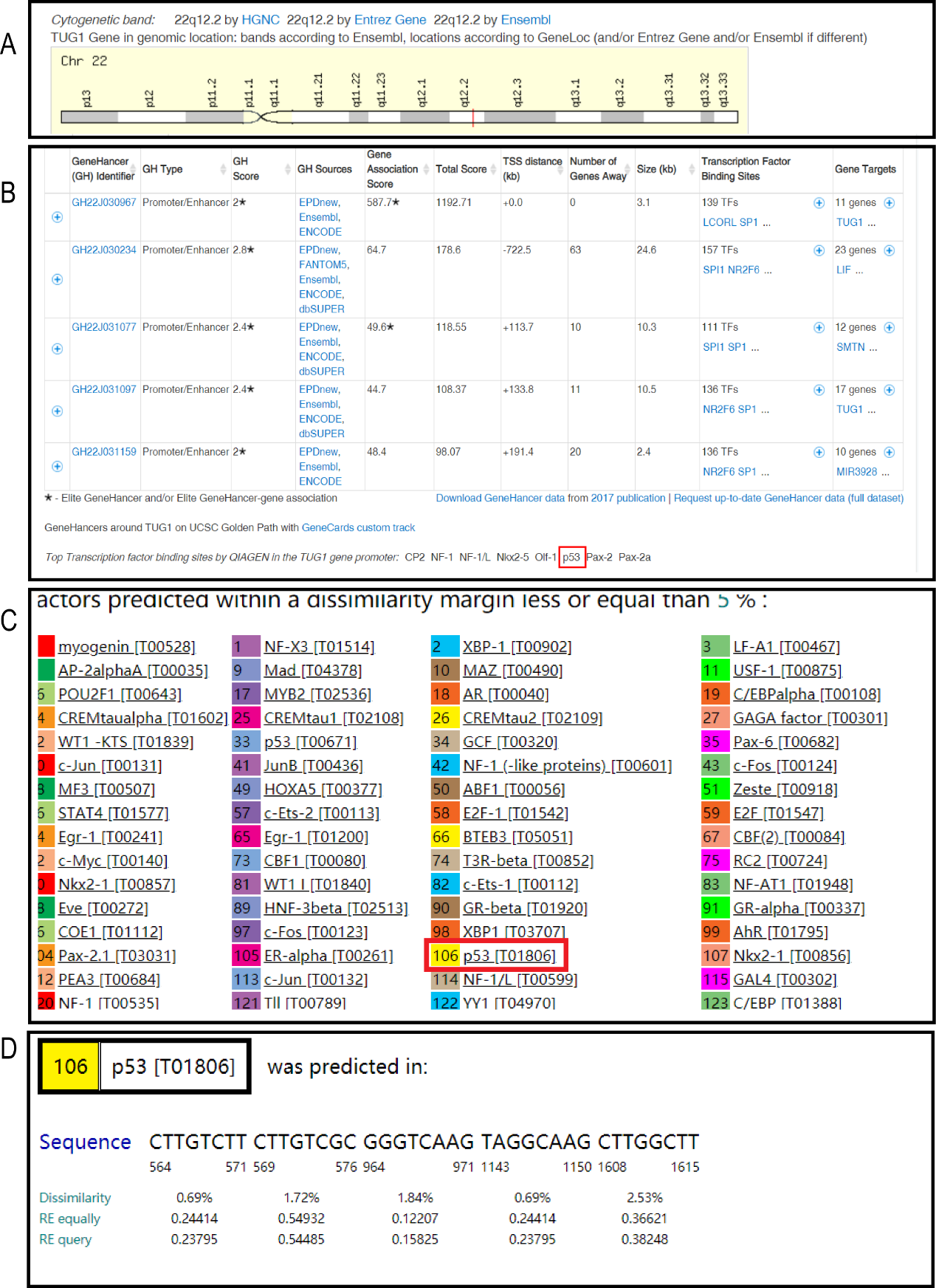


**Supplementary Figure 1. P53 is a transcription factor of TUG1. (A):** GeneCards database shows the location of TUG1 in genomic. **(B)**: GeneCards database shows the gene Hancer Regulatory Elements, and p53 is one of the top transcription factors can band to TUG1 promoter (red box marked). **(C)**: Promo database shows the p53 is one of the predicted transcription factors of TUG1(red box marked). **(D)**: Promo database shows that p53 banding site in TUG1 promoter.
