# Supplementary material for: P53 and taurine upregulated gene 1 promotes the repair of the DeoxyriboNucleic Acid damage induced by bupivacaine in murine primary sensory neurons
Source: Bioengineered. 2022 Mar 10;13(3):7439–56. doi: 10.1080/21655979.2022.2048985 (PMC9208530; doi:10.1080/21655979.2022.2048985)
Supplement: Supplemental Material [file KBIE_A_2048985_SM0069.zip › Supplenment Table 1.docx]

supplenment table 1

| TUG1 promoter |
| --- |
| TGCAGCTCGCAGATGCCTTTGTCCCTCCCCGTGGAATTGGAACTCAACCACCGCAGCGCGCTCTTGGAACCATGTGCTGCCTCCGCTGCCGCTGCCGCCGCCGCCGCCGCCGCCCGCCCGCGCCGCGACACGACACGACACGCCGCGCCTCAACAGACGATGACAGGCTGGGCGTCACGAGATTCAGCGTTCAGTACGGCTTTGCGGCCGGTGCTGCCCGCGCCACCGCTACCTCAGCCACCGCTTTGTCTCCGTAAGCGCACGCAGCGCGAATCACCTCGCTCACTCCCGACGGCCCGGCCGCCGAGGCCTCCAGCGCACCTTCGGCCAATCACAGAGCTCGAAGTCTTCCCGCCCCCGCCCTGAGACTCCACCTTCTGATTGGCGAGCGACGTCGGTCCACGTGACCGGATCTTGTTTGGCCGGTCCGCCCCCATCACGTGAGGGCGCGCGCCTCTCTTCCTGCTTTCCTGACCCCTCTCGGCCATTTAAAGGAACAGTACCGGGGGCGGGCCGAGAGACGCAGCCGGGACGGTAGCTGCAGAGCAGAGCGGAGGAGCCATCTTGTCTTGTCGCCGGGGAGTCAGGCCCCTAACTCGAAGAAGCCCTGGCGCGCCCTCCCCCCCTCCCCGGTCTGGTAGGGCGAAGGAGCGGGCGTGCGGTCGATCGAGCGATCGGTTGGCGGCTCTTTCTCCTGCTCTGGCATCCAGCTCTTGGGGCGCAGGCCCGGCCGCCGCGGCGCGCGCCCGGTGGCCGTTGGCGCTCGCGCCGCGTCTTTCTTCTCGTACGCAGAACTCGGGCGGCGGCCTATGCTTTTGCGATCCGACGAGGGGTCGTCCGGGTGGTTGGCGGCGGCGGGCAACTCCGCCCCGCTCCCGGGGAGGCGGCGGGGGAAGCTGGGGTGGCCGGGGCTGGCCTGGAGGCCTGGCGCCACCCCTCGGGGCCTGCTAGGACCCAGTTGGAGGGTCAAGAGGGAGCTGGAGGATGGTTGGTGGTGGGCTTCCTCCTTTGCCTTTTCCTACTTATGCCACCTTAGTGGGGAGCGAGAGCGCTGGCGGCAGCTCCGGGGTGGGGATTCGAGCTCCGGAGTCGGAAGATCTGGGTTTGCTTCCGGGCCTAGCCACTCGCTGGCCGAGTGACCTTAGGCAAGTCACTCTGTAATCTGTCTGCGCCTCAGTTTCCTCCTCTGCCTATCAGTGTGTGTATGGAATTGGAAGCTCTCTTCAAAAGTGTCGGTTTACAGAAAGATGATTATTGTTTTTTTTTCCTTTGAGTTGCAAGAAGTCATAACAATTGCTCATTCCCTGAAATTAAATTCGGGAACCTTTTATTGGAGTTGGAGATGGGAAGGTTCCCTGAACTTCTTCTGTACAGAAACACGTGGGAAAGTGTTTTCTAGGGGGATGAGTAGATCCTTCAGTAGCGAGTTTTCAAAGGGGGCTATGACCCAGAAGAGTTTAGAACAGTTTTTGCCACCGGGAGGAAACTGAGGCCTACATGTCATCTGGGACCCTTCACAACCAATATGAAGCCCCCATTTGAGTCCCTGGTATATGTGAGCTGCTTCTTACTGCATAATGGCTGTTTCAGGTACCATCACTTCACTGCTTGGCTTTTAGTCAAAGCCCTTTCCTTTCACCATAGGGTGCCTGAAGGGTGGCTGATGCCTATGGTACAATGGCACCCACTAGAAAGCAGCTACAATTAGGAGTGAATGTGTCCTCGAGTAACAAGAAGTGTAATCTCTGGCCCAGCGATTGTTTTCTGCTGCTCGTACTGGTGCCTGACTACTTTTCTCTCGTTAACTATTTTTTTACAACCATGATTGACACCCATTATTTGCTCCTATTTTATATCCCTGGTTTTCGTGGTTGATAGTGAACAGGATTATTTTTAAATGGTGCTGCCAAAACCAGTTAATAGTGCACATGGATTTTTAGCAAGAGGGCTACTTGGAACAGACTGAACCAGGCGTTGGATTCCTGAAAATGTTTGGGGTT |
